# Do We Really Need Expensive Stent-Grafts to Treat External Iliac Artery Lesions Effectively?

**DOI:** 10.1007/s00270-023-03430-0

**Published:** 2023-04-06

**Authors:** Osman Öcal, Max Seidensticker

**Affiliations:** Klinik und Poliklinik für Radiologie, Klinikum der Universität München, LMU, Marchioninistr 15, 81377 Munich, Germany

## Introduction

We read the work of Squizzato et al. with great interest [1]. They reported a multicenter retrospective cohort of 93 patients with steno-occlusive lesions at the external iliac artery (EIA) treated using self-expandable covered stent (Viabahn, W.L Gore & Associates, Inc., Flagstaff, AZ-USA). Most treated patients had advanced disease, with 72% having TASC (Trans-Atlantic Inter-Society Consensus Document on Management of Peripheral Arterial Disease) II C or D category lesions. There was calcification covering more than 50% of vessel circumference in 30.1% of the patients. They reported an excellent technical outcome with 100% technical success with no procedural death. The median follow-up was 25 months, the primary patency rate at 42 months was 89.8%, and the limb salvage rate was 94.6%.

Currently, Society for Cardiovascular Angiography and Interventions (SCAI) guidelines on device selection recommend balloon-expandable covered stents (IIa, moderate recommendation) over self-expandable grafts (IIb, weak) in focal EIA lesions, both with the same class in diffuse EIA lesions with IIa (moderate) [2]. However, as the initial treatment device, self-expandable bare metal stents (BMS) are recommended for both lesions (I, strong). On the other hand, in case moderate (≥ 180 and < 1/2 of vessel length) or severe (≥ 180 and > 1/2 of vessel length) calcification, balloon-expandable covered stents are recommended (I, strong) over self-expandable covered stents (IIa, moderate) and BMS (IIa, moderate).

A randomized controlled trial evaluating covered and BMS for the aortoiliac occlusive disease has compared a balloon-expandable covered stent (Advanta V12, Atrium Medical Corp, Hudson, NH-USA) with BMS using a primary outcome parameter of binary stenosis (≥ 50) at the 18th month [3]. Covered stents had a significantly lower rate of restenosis (HR, 0.35; 95% CI, 0.15–0.82; *p* = 0.02), but there was no significant difference in complete occlusion rate (HR, 0.28; 95% CI 0.07–1.09, *p* = 0.07). Subgroup analyses have shown no difference between covered and BMS in TASC II B lesions, while covered stents had significantly lower restenosis in TASC II C and D lesions. This result led to the aforementioned recommendation of balloon-expandable covered stents in moderate or severely calcified lesions. A meta-analysis of four studies, including this trial, has shown no significant difference in the primary patency rate between covered and BMS (85.9 vs. 80.4%, pooled OR 2.10, 95% CI 0.48–9.11, *p* = 0.32) [4]. And the recently published DISCOVER trial comparing balloon-expandable covered and BMS has also failed to meet the primary outcome of binary stenosis [5]. At two years, the lack of > 50% stenosis rate was 89.7% in the covered stent arm and 83.3% in the BMS arm (*p* = 0.24). Although these studies were not focused only on the external iliac artery as Squizzato et al. did, these results show that using covered stents does not provide a clear benefit in terms of patency.

The main idea behind the potential advantage of covered stents is limiting neointimal hyperplasia, and eventually, restenosis by excluding underlying atherosclerotic plaque. However, this benefit comes with the expense of delayed endothelization by interfering with smooth-muscle migration which may require longer dual antiplatelet treatment. Additionally, covered stents have larger delivery systems which carry additional risk for access complications. Furthermore, covered stents cost considerably more than BMS. For example, the device evaluated by Squizzato et al. costs approximately 2700€ in Germany, a balloon-expandable covered stent around 1600€, while a BMS (i.e., E-Luminexx, Bard/Angiomed, Karlsruhe, Germany) costs only around 400€. Considering also the need for multiple devices in long lesions (the mean number of the device was 1.1. + 0.3 in this study) this cost difference will increase. Taking these downsides into account, before covered stents become the routine first choice in patients with aortoiliac occlusive disease, high-quality data showing clear clinical benefit over BMS are needed, especially in patients without moderate or severely calcified lesions.
